# Impact of Diagnosis-Related-Group (DRG) payment on variation in hospitalization expenditure: evidence from China

**DOI:** 10.1186/s12913-023-09686-z

**Published:** 2023-06-24

**Authors:** Qiaosheng Li, Xiaoqi Fan, Weiyan Jian

**Affiliations:** Department of Health Policy and Management, School of Public Health, Peking University, 38 Xueyuan Road, Haidian District, Beijing, China

**Keywords:** China, Diagnosis-Related-Group (DRG), Hospitalization expenditure variation, Medical care service consistency, Payment reform

## Abstract

**Background:**

Diagnosis-Related-Group (DRG) payment is considered a crucial means of addressing the rapid increases of medical cost and variation in cost. This paper analyzes the impact of DRG payment on variation in hospitalization expenditure in China.

**Method:**

Patients with chronic obstructive pulmonary disease (COPD), acute myocardial infarction (AMI) and cerebral infarction (CI) in a Chinese City *Z* were selected. Patients in the fee-for-service (FFS) payment group and the DRG payment group were used as the control group and intervention group, respectively, and propensity-score-matching (PSM) was conducted. Interquartile distance (IQR), standard deviation (SD) and concentration index were used to analyze variation and trends in terms of hospitalization expenditure across the different groups.

**Results:**

After DRG payment reform, the SD of hospitalization expenditure in respect of the COPD, AMI and CI patients in City *Z* decreased by 11,094, 4,833 and 4,987 CNY, respectively. The concentration indices of hospitalization expenditures for three diseases are all below 0 (statistically significant), with the absolute value tending to increase year by year.

**Conclusion:**

DRG payment can be seen to guide medical service providers to provide effective treatment that can improve the consistency of medical care services, bringing the cost of medical care closer to its true clinical value.

## Introduction

Consistency in medical care service is an important indicator of medical quality, safety and even equity [1, 2]. Poor consistency arises from differences in medical care. If the variation in medical care service provision is large, then it is likely to be the case that many patients are not being treated according to the standard clinical disease treatment guidelines [3, 4]. Variation in medical care service is often reflected in variation in medical costs, so many researchers use it as an indicator to analyze the consistency of medical care service. A study conducted in the United States by Elliot Wakeamet al*.* [5] analyzed hospitalization expenditure of patients undergoing five different kinds of operations, and found that the distribution range of hospitalization expenditure was more than 10 times higher than the expected cost range evaluated by standard clinical guidelines. In another American study, Raymondet al*.* [6] report that, in respect of patients treated for lung cancer in different hospitals, the difference in total cost could be greater than 12,000 USD. Meanwhile, a Japanese study on medical costs incurred by patients with similar neurological diseases showed that, even in the case of diseases with similar mechanism of action, pathogenesis and treatment processes, the cost difference between patients could be up to three times or more [7]. Such high variation in medical costs is the strong evidence that medical service providers have failed to provide consistent services to patients during diagnosis and treatment, which might result in additional economic burden being imposed on patients in a way that damages the quality of medical care and health equity [8–10].

It can be observed that medical costs in China have increased at a very high rate and in a way that has also given rise to significant variation in medical costs. Between 2007 and 2012, China’s real per capita health expenditure grew by 14.9% annually, with per capita GDP growing by only 10.2% [11]. The results of a study on the medical costs of inpatients in a tertiary hospital in a developed eastern city of China show that, from 2009 to 2017, the differences in the costs of patients with the same diseases gradually expanded [12]. Another study conducted in patients with new rural cooperative medical insurance (NRCMI) in an underdeveloped area of central China shows that, from 2006 to 2014, the hospitalization expenditure in county-level hospitals increased by nearly 41%, from 2,988 CNY to 4,200 CNY [13]. Researchers have also analyzed changes in Chinese medical costs by using data from the China Health Statistical Yearbook from 2002 to 2006, and found yearly increases in variation in both outpatient and inpatient expenses, mainly due to the rapid rise of costs associated with drugs and materials [14]. Fee-for-service (FFS) payment is considered an important reason of such rapid growth in medical costs and their variation. Healthcare providers have a strong tendency to induce demand in a way that results in overuse of medical services [15] in FFS payment, so treatments that patients receive deviate very clearly from clinical guidelines [16].

At present, many low-and middle-income countries (LMICs) are actively introducing Diagnosis-Related-Group (DRG) payment as a means of controlling medical costs and helping more patients to receive standardized medical care service. Since 2010, DRG payment pilot programs have been carried out in a number of cities in China. Theoretically, under the DRG payment model, doctors are more motivated to implement necessary treatment in accordance with clinical guidelines, which will have an impact on the incentive to save on medical costs [17, 18], inhibiting induced demand on the part of the provider and thereby decreasing medical costs and variation. Such effects can be witnessed in the practices of more economically advanced countries that have introduced DRG payment. For example, since Germany introducing DRG payment in 2004, the growth rate of per capita medical expenses has gradually decreased, the variation has narrowed and the proportion of out-of-pocket payment (OOP) in terms of total medical expenses has also gradually declined. In the United States, the introduction of DRG payment was accompanied by a decrease in total hospitalization expenditure, the proportion of OOP [19], and non-essential additional expenditures [20].

This paper aims to analyze the impacts of DRG payment on variation in medical costs in China, and evaluate the role of DRG payment system design in regulating medical care service. Consequently, practical evidence will be provided for other LMICs introducing models grounded in DRG payment.

## Methods

### Samples

City *Z* began to implement DRG payment reform in 2012. Six tertiary first-class public hospitals were selected as the pilot, in which all discharged cases were in DRG payment, while the remaining hospitals were still in FFS payment. In addition, during the period 2010–2016 that this study focused on, there were no other policies implemented that might affect hospitalization expenditure. The data were collected from a medical insurance database relating to policyholders of the urban employee basic medical insurance (UEBMI) in City *Z*. These data included information on variables such as patients’ basic demography (age and gender), primary diagnosis, secondary diagnosis, and hospitalization expenditures (drug expenditure, materials expenditure, total expenditure). The reform of this city’s DRG payment system was implemented in 2012. In order to avoid being influenced by the introduction of another policy affecting medical expenses, which was named *the cancelling of drug mark-up costs* issued in 2017, only data from 2010 to 2016 were considered. In terms of how this seven-year period can be broken down, 2010–2011 was the period before reform of the DRG payment, and the period 2012–2016 represents the situation following reform in DRG payment. In terms of research objects, we select patients with chronic obstructive pulmonary disease (COPD), acute myocardial infarction (AMI) and cerebral infarction (CI) to reflect the variation in hospitalization expenditure for both internal and surgical diseases on the basis that these conditions have clear clinical guidelines, feature in a high number of cases, and tend to incur high medical costs (with ICD-10 codes of J44.0, I21.0 and I63.0, respectively). The Charlson Comorbidity Index (CCI) was used to evaluate the severity of each given patient’s condition, with CCI ≤ 1 indicating a low level of severity. All cost data were used to eliminate the impact of inflation through the urban consumer price index (CPI) given on the official website of the Chinese Bureau of Statistics.

### Intervention group and control group

Six hospitals in City *Z* have implemented DRG payment, and all other medical institutions in this city continue to operate using pay on FFS payment. The intervention group was taken to comprise patients with COPD, AMI and CI in the six former hospitals, while the control group was taken to comprise patients from the other hospitals. The intervention group and the control group were matched by 1:1 propensity-score-matched (PSM) year after year. To exclude the influence of individual differences on the outcome, the propensity score was obtained by logistic regression of the patients’ age, gender, and CCI. The Caliper value of the matching accuracy was 0.02.

### Statistics

The study was conducted to compare the variation in the trend of hospitalization expenditure in respect of the intervention and control groups before and after the implementation of the DRG payment. The degree of variation was taken to be represented by the interquartile range (IQR) and standard deviation (SD). The smaller the value, the more concentrated the distribution of hospitalization expenditures. The calculation formulas of IQR and SD are shown in (1) and (2).

1 $$IQR=\left({Q}_{3}, { x}_{.75}\right)-\left({Q}_{1}, {x}_{.25}\right)$$

2 $$SD=\sqrt{\frac{{\sum }_{i=1}^{n}{\left({x}_{i}-\overline{x }\right)}^{2}}{n-1}}$$

*Q*_3_, *x*_.75_ *and* *Q*_1_, *x*_.25_ respectively represent the hospitalization expenditure of the third and first quartile, $\overline{x }$ represents the average hospitalization expenditure, and n represents the number of samples.

Concentration index was selected as an indicator as a reflection of the distribution of hospitalization expenditure in respect of the different groups of patients. Meanwhile, the Kruskal–Wallis test was used to compare the concentration indices across years and *P* values were calculated. The calculation formula for concentration index is shown in (3).

3 $$\mathrm{concentration}\;\mathrm{index}=\frac{2cov\left(h_i,r_i\right)}{\overline x}$$

$cov\left({h}_{i},{r}_{i}\right)$ represents the covariance matrix of each patient’s hospitalization expenditure (${h}_{i}$) and each patient’s grade in the sample (${r}_{i}$). The matter of whether it is intervention group or not is selected as the grade variable (${r}_{i}$=1, intervention group; ${r}_{i}$=0, control group) to reflect the degree of concentration of hospitalization expenditure in different grades. The absolute value of concentration index reflects the degree of concentration: The closer it is to 0, the more uniform the distribution of hospitalization expenditures is; conversely, the more concentrated the distribution is. If the value is less than 0, this suggests that hospitalization expenditure was more concentrated in the intervention group; if the value is greater than 0, it was more concentrated in the control group.

To further explore the sources of the variance of expenses in the different groups, analysis was carried out on the composition of hospitalization expenditure and the proportion of each component, including Drug Expenditure, Material Expenditure and Other Expenditure.

The significance level of the statistical tests was set such that $\alpha =0.05$. All statistical analyses were performed using Stata SE 15.0.

## Results

### Basic information on samples

From 2010 to 2016, there were 14,094 patients with COPD, 17,897 patients with AMI and 26,063 patients with CI in the database. 6,238 patients with COPD, 11,086 patients with AMI and 14,906 patients with CI were included in the study after PSM, and the control and intervention groups were equally divided. Table 1 provides information relating to the distribution of patients with different disease types after PSM in the two different groups. More than twice as many men as women can be seen to have had COPD. Around 90% of COPD patients were over 60 years old, and more than 80% of patients had a less severe form of the disease. The number of male AMI patients is more than four times the number of women. More than 90% of AMI patients were between 40 and 79 years old and more than 82% of AMI patients had less severe forms of the disease. About three times as many men as women can be seen to have suffered from CI, thereinto, more than 80 percent of patients were between 40 and 79 years old, and more than 50 percent of patients had severe forms of the disease. The distribution of sex, age and CCI were consistent between both control and intervention groups after PSM.

**Table 1:** Characteristics of samples

Diseases	Variables		Match Group		Intervention Group	
			N=	%	N=	%
**COPD**	**Sex**	Male	2172	69.64	2160	69.25
		Female	947	30.36	959	30.75
	**Age**	18–39	3	0.10	2	0.06
		40–59	329	10.55	340	10.90
		60–79	1966	63.03	1983	63.58
		≥ 80	821	26.32	794	25.46
	**CCI**	0	1279	41.01	1245	39.92
		1	1300	41.68	1311	42.03
		≥ 2	540	17.31	563	18.05
	**Count**		**3119**	**100**	**3119**	**100**
**CI**	**Sex**	Male	5538	74.31	5447	73.08
		Female	1915	25.69	2006	26.92
	**Age**	18–39	161	2.16	151	2.03
		40–59	2531	33.96	2527	33.91
		60–79	3977	53.36	3994	53.59
		≥ 80	784	10.52	781	10.48
	**CCI**	0	649	8.71	403	5.41
		1	3062	41.08	3225	43.27
		≥ 2	3742	50.21	3825	51.32
	**Count**		**7453**	**100**	**7453**	**100**
**AMI**	**Sex**	Male	4461	80.48	4456	80.39
		Female	1082	19.52	1087	19.61
	**Age**	18–39	130	2.35	162	2.92
		40–59	2450	44.20	2474	44.63
		60–79	2633	47.50	2587	46.67
		≥ 80	330	5.95	320	5.77
	**CCI**	0	2335	42.13	2293	41.37
		1	2252	40.63	2286	41.24
		≥ 2	956	17.25	964	17.39
	**Count**		**5543**	**100**	**5543**	**100**

Table 2 shows the composition and proportion of hospitalization expenditure in respect of the intervention group and the control group. In relation to COPD and CI patients, the cost of drugs accounted for greatest proportion (around 50%), and the cost of material accounted for the least (around 10%). Among AMI patients, materials accounted for the greatest proportion (more than 70%). In both the control and intervention groups, the distribution of hospitalization expenditures was consistent.

**Table 2:** Composition of hospitalization expenses of patients

Diseases	Group	Drug Expenditure		Material Expenditure		Other Expenditure	
		**CNY**	**%**	**CNY**	**%**	**CNY**	**%**
**COPD**	Matched Group	7937.14	52.06	1513.26	9.92	5798.31	38.02
	Intervention Group	7423.55	52.47	1459.50	10.32	5264.79	37.21
**CI**	Matched Group	9550.41	49.19	2012.43	10.36	7854.91	40.45
	Intervention Group	7184.32	50.41	1221.47	8.57	5846.70	41.02
**AMI**	Matched Group	5386.27	8.55	46355.30	73.58	11254.61	17.87
	Intervention Group	4649.69	8.51	39928.32	73.09	10052.18	18.40

### Trends in SD of hospitalization expenditure

Table 3 shows the SD in hospitalization expenditure of the intervention and control groups as well as the change in the last year. It also provides the Double-Difference Value representing the annual change difference between the intervention group and the control group.

**Table 3:** The SD and double difference value of hospitalization expenses of patients (CNY)

	**Control Group**		**Intervention Group**		**Double-Difference Value**
	**SD**	**Change**	**SD**	**Change**	
**COPD**					
**Before (FFS payment only)**					
2010	17536.87	——	22798.05	——	——
2011	15337.87	-2199.00	17587.92	-5210.13	-3011.13
**After (DRG payment in pilot hospitals)**					
2012	10215.21	-5122.67	6493.26	-11094.66	-5971.99
2013	7037.40	-3177.81	5778.24	-715.02	2462.78
2014	9009.47	1972.07	5789.14	10.91	-1961.16
2015	13955.59	4946.12	6538.47	749.32	-4196.79
**AMI**					
**Before (FFS payment only)**					
2010	28310.83	——	26213.95	——	——
2011	27514.44	-796.39	24327.57	-1886.38	-1089.99
**After (DRG payment in pilot hospitals)**					
2012	25165.85	-2348.59	19495.07	-4832.50	-2483.91
2013	26146.35	980.50	16363.33	-3131.73	-4112.24
2014	26776.27	629.92	18002.29	1638.96	1009.04
2015	28379.07	1602.80	18546.57	544.28	-1058.52
2016	28120.67	-258.40	20323.44	1776.87	2035.27
**CI**					
**Before (FFS payment only)**					
2011	15755.92	——	13571.37	——	——
**After (DRG payment in pilot hospitals)**					
2012	13612.52	-2143.39	8583.88	-4987.49	-2844.09
2013	16126.07	2513.55	8159.43	-424.45	-2938.00
2014	11558.56	-4567.51	6681.67	-1477.76	3089.75
2015	12238.64	680.08	6076.18	-605.49	-1285.56
2016	14112.71	1874.07	5967.37	-108.82	-1982.89

In relation to COPD patients, following the implementation of DRG payment, the SD of hospitalization expenditures in the intervention group was lower than that of the control group for every year. The SD of hospitalization expenditure for the intervention group decreased the most in the first year following reform to the DRG payment policy (2012), being 5972 CNY more than that in the control group.

In relation to AMI patients, following the DRG payment policy reform, the SD of hospitalization expenditures in the intervention group decreased by 2,483 CNY more than that of the control group. And, in 2013, the magnitude of the decrease grew to 4,112 CNY more than the control group. Although the SD of hospitalization expenditure in the intervention group increased year on year from 2014 to 2016, the absolute value was still smaller than that of the control group.

Finally, in relation to CI patients, there was no corresponding main diagnosis in the database in 2010, and only the results from 2011 to 2016 could be considered. It can be seen that the SD of hospitalization expenditures in the intervention group continued to decline following the DRG payment policy reform, with all decreasing more than the control group in other years except 2014.

### Trends in interquartile range of hospitalization expenditures

**Fig. 1:**
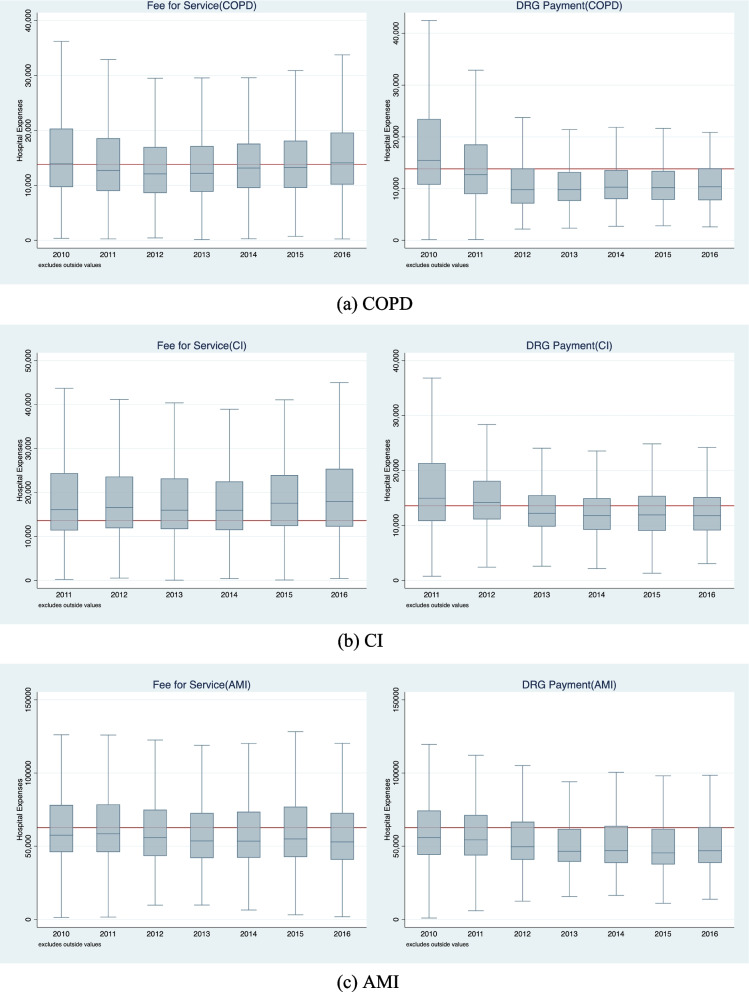
Box chart of hospitalization expenses of patients

Figure 1 shows the IQR, upper adjacent value and lower adjacent value of hospitalization expenditure in relation to patients. The red line represents the fixed payment amount for each DRG.

For COPD patients, after the DRG payment reform, the IQR of the intervention group can be seen to be smaller than that for the control group in each year. The declining trend of the IQR was the most obvious in the first year after the reform, and the median of hospitalization expenditure can be seen to be lower than the fixed level of DRG payment. In the control group, there is no obvious decreasing trend in terms of the IQR, and there is little difference between the median hospitalization expenditure and the fixed amount, which is higher than the intervention group.

For CI patients, the IQR of hospitalization expenditure in the intervention group decreased significantly following the DRG payment reform, and the IQR in all years can be observed to be smaller than those relating to the control group. From 2013, the median hospitalization expenditure is lower than the fixed level of DRG payment. For the control group, the IQR of hospitalization expenditure shows no downward trend, and the median hospitalization expenditure is always higher than the fixed level of DRG payment.

For AMI patients, following the DRG payment reform, the IQR of hospitalization expenditure for the intervention group is smaller than that of the control group, most markedly in 2012 and 2013. The median expenditures of the two groups are both lower than the fixed level of DRG payment. The median hospitalization expenditure of the intervention group is lower than that of the control group and demonstrate a continuous downward trend.

### Concentration index of hospitalization expenditure

Table 4 shows the concentration index of patients’ hospitalization expenditure. In the first year following the reform of DRG payment in 2012, the concentration indices of hospitalization expenditure for three diseases all change to below 0, with statistical significance. And every year after 2012 the concentration indices are all below 0. The changes in concentration index of hospitalization expenditure for COPD patients are statistical significance in 2014 and 2016, with the absolute value gradually increasing from 2012 to 2015. The changes in concentration index of hospitalization expenditure for CI patients are all statistically significant, with the absolute value increasing year by year. The changes in concentration index of hospitalization expenditure for AMI patients are statistically significant in 2013.

**Table 4:** The concentration index of hospitalization expenses of patients

Year	COPD	CI	AMI
**Before (FFS payment only)**			
2010	0.035	——	-0.012
2011	0.009 ^**^	-0.038	0.020
**After (DRG payment in pilot hospitals)**			
2012	-0.045 ^***^	-0.048 ^***^	-0.026 ^***^
2013	-0.044	-0.067 ^***^	-0.036 ^***^
2014	-0.049 ^**^	-0.067 ^*^	-0.034
2015	-0.052	-0.069 ^***^	-0.045
2016	-0.030 ^***^	-0.114 ^***^	-0.023

The *P* value of the Kruskal–Wallis test reflects the change in the concentration index compared to the previous year, * *P* < 0.05, ** *P* < 0.01, *** *P* < 0.001

## Discussion

The results of this study show that, variation in hospitalization expenditures of both internal diseases (represented by COPD and CI) and surgical diseases (represented by AMI) decreased year by year after the implementation of the DRG payment system, while variation in hospitalization expenditures in FFS payment are witnessed to be significantly higher during the same period. This suggests that China’s DRG payment reform can motivate doctors to provide more standardized medical care service in a manner that may have contribution to reduce variation in medical care costs and improving the consistency of medical care service.

Previous studies have shown that doctors’ behavior is a key factor in variation in medical costs. The average cost of a single type of surgical operation varies among different surgeons [21], and the variation between doctors is greater than that between hospitals [22]. It is on this basis that doctors’ behavior can be isolated as a major factor for explaining variation in medical costs. Other studies provide evidence that doctors’ preferences in diagnosis and treatment can reduce variation in hospitalization expenditure to a greater extent than the severity of the given disease itself [23] and that the reduction in hospitalization expenditure variation is more obviously attributable to the standardization of doctors’ behaviors [23, 24].Besides, the payment methods associated with medical insurance is regarded as the “key” to guide the behavior of medical service providers [25, 26]. Under the DRG payment model, the profits associated with drugs and materials, previously paid by FFS, become costs [27], and the financial risks caused by overspending on medical expenses are shifted from insurers to medical service providers, who take on a more active role in controlling costs [28]. On this approach, the main method of reducing cost overruns is to perform treatment in a symptom-centered way, carrying out diagnoses and treatment activities strictly in accordance with the standard clinical pathways and making efficient and reasonable use of medical resources [29, 30].

Previous studies from developed countries have also provided evidence that variation in hospitalization expenditure decreases following the implementation of DRG payment, thus providing supporting evidence for the conclusions drawn in this study. In the United States, McMahon L and Newbold R [23] used a Maryland inpatient medical insurance database to analyze differences in variation in hospitalization expenditure for 10 diseases between DRG and non-DRG payment medical institutions and found that variation in hospitalization expenditure was small under the former (DRG-based) model. They also found that the most important factor affecting variation in hospitalization expenditure was doctors’ behavior in diagnosis and treatment. Using data from the medical service survey conducted by the American Hospital Association in 2005, Huerta Tet al*. *[31] analyzed differences in the efficiency of different medical institutions and found that in hospitals operating using DRG payment, variation in expenditure was low and that medical efficiency and costs were negatively correlated, demonstrating a link between high costs and low efficiency of medical service.

The present study also found that patients with AMI were associated with the smallest decrease in the degree of variation in terms of hospitalization expenditure both before and after DRG payment reform. This can be explained in terms of the point that the treatment of AMI is now relatively mature as a medical procedure, accompanied by an industry consensus and universal clinical guidelines as a means of standardizing doctors’ behaviors [32]. It is on this basis that it is possible to explain why the impact of DRG payment on the degree of variation on hospitalization expenditure of AMI patients is relatively weaker than other groups. It could also be estimated that standardized clinical guidelines can provide guidance relevant to ensuring consistency in doctors’ behavior and have some influence on reducing variation in medical care costs [33]. In addition, according to the analysis of the composition, the majority of hospitalization expenditures in the internal medicine group are drug costs, while those in the surgical group are materials costs. This suggests that, in the future, the use of DRG in the internal medicine group and the surgical group can lead to greater standardization of the treatment process through an increased focus on standardized drug use and materials use in the formulation of clinical guidelines for different types of DRG. Core to this approach is the goal of making reasonable use of medical resources and providing effective medical care service [34, 35].

The findings of this study illustrate the effectiveness and advantages of the DRG payment reform, and fully demonstrate its necessity. Most importantly, this study can provide convinced practical evidence and experience for LMICs which are trying to reform payment system to address the rapidly rising of medical care costs.

### Limitations

There are two main limitations to highlight. First, due to the nature of the data, it is not possible to describe which specific services might have changed during the treatment process. Second, given the lack of data on quality of care, it is unclear whether the reductions in cost variability highlighted are also accompanied by a change in quality of care. These are points of interest that might be taken up in further studies.

## Conclusion

The Chinese data considered in this study provide evidence that DRG payment can help reduce variation in medical care costs, suggesting that providers are more likely to follow clinical guidelines and conduct effective treatment after taking the risk of overspending, with DRG payment bringing the cost of care closer to its true clinical value in a way that is not observed for FFS. This practical evidence from China gives confidence to other LMICs that seek to introduce DRG payment reform and proves advantages of DRG payment, which can help them design more scientific and efficient payment system. It should be borne in mind, however, that even with the introduction of DRG payment, quality monitoring will be necessary to prevent providers going to the other extreme of squeezing costs to the detriment of healthcare quality.

## Data Availability

The datasets analyzed during the current study are not publicly available due to information security and confidentiality requirements, but are available from the corresponding author on reasonable request.

## Abbreviations

DRG: Diagnosis-Related-Group
FFS: Fee-for-service
LMIC: Low-and middle-income country
COPD: Chronic obstructive pulmonary disease
CI: Cerebral infarction
AMI: Acute myocardial infarction
IQR: Interquartile range
SD: Standard deviation
UEBMI: Urban employee basic medical insurance
NRCMI: New rural cooperative medical insurance
OOP: Out-of-pocket payment
CPI: Consumer price index
CCI: Charlson Comorbidity Index
PSM: Propensity score matching
